# Identification of *TRPC6* as a possible candidate target gene within an amplicon at 11q21-q22.2 for migratory capacity in head and neck squamous cell carcinomas

**DOI:** 10.1186/1471-2407-13-116

**Published:** 2013-03-14

**Authors:** Sandra Bernaldo de Quirós, Anna Merlo, Pablo Secades, Iriana Zambrano, Ines Saenz de Santa María, Nerea Ugidos, Eloisa Jantus-Lewintre, Rafael Sirera, Carlos Suarez, María-Dolores Chiara

**Affiliations:** 1 Servicio de Otorrinolaringología, Hospital Universitario Central de Asturias, Instituto Universitario de Oncología del Principado de Asturias, Universidad de Oviedo, Oviedo, Spain; 2 Laboratorio Oncología Molecular, Fundación para la Investigación del Hospital General Universitario de Valencia, Valencia, Spain; 3 Departamento de Biotecnología, Universidad Politécnica de Valencia, Valencia, Spain

**Keywords:** Head and neck squamous cell carcinoma, TRPC6, Invasion, Gene amplification

## Abstract

**Background:**

Cytogenetic and gene expression analyses in head and neck squamous cell carcinomas (HNSCC) have allowed identification of genomic aberrations that may contribute to cancer pathophysiology. Nevertheless, the molecular consequences of numerous genetic alterations still remain unclear.

**Methods:**

To identify novel genes implicated in HNSCC pathogenesis, we analyzed the genomic alterations present in five HNSCC-derived cell lines by array CGH, and compared high level focal gene amplifications with gene expression levels to identify genes whose expression is directly impacted by these genetic events. Next, we knocked down *TRPC6*, one of the most highly amplified and over-expressed genes, to characterize the biological roles of *TRPC6* in carcinogenesis. Finally, real time PCR was performed to determine *TRPC6* gene dosage and mRNA levels in normal mucosa and human HNSCC tissues.

**Results:**

The data showed that the HNSCC-derived cell lines carry most of the recurrent genomic abnormalities previously described in primary tumors. High-level genomic amplifications were found at four chromosomal sites (11q21-q22.2, 18p11.31-p11.21, 19p13.2-p13.13, and 21q11) with associated gene expression changes in selective candidate genes suggesting that they may play an important role in the malignant behavior of HNSCC. One of the most dramatic alterations of gene transcription involved the *TRPC6* gene (located at 11q21-q22.2) which has been recently implicated in tumour invasiveness. siRNA-induced knockdown of *TRPC6* expression in HNSCC-derived cells dramatically inhibited HNSCC-cell invasion but did not significantly alter cell proliferation. Importantly, amplification and concomitant overexpression of *TRPC6* was also found in HNSCC tumour samples.

**Conclusions:**

Altogether, these data show that *TRPC6* is likely to be a target for 11q21–22.2 amplification that confers enhanced invasive behavior to HNSCC cells. Therefore, *TRPC6* may be a promising therapeutic target in the treatment of HNSCC.

## Background

The broad application of cytogenetic and molecular genetics methods has led to the identification of tumor-associated chromosomal regions substantial for the tumorigenesis and progression of head and neck squamous cell carcinomas (HNSCC) [1-3]. Comprehensive analysis of recurrent amplified chromosomal regions has allowed identification of oncogenes and other cancer-related gene such as *EMS1*, *CCND1*, *PPFIA1*, *TAOS1* (11q13), *LOXL4* (10q24), *PAK4* (19q13), and *HIF1A* (14q23-q24) which have been associated with different clinical behaviors [4-10]. Therefore, associations of high-level genomic amplifications with altered gene expression and functional analysis of the affected genes represents an excellent approach to identify novel genes involved in tumor progression and carcinogenesis.

Here, we compared the genome-wide DNA copy number alterations present in five HNSCC-derived cell lines with those previously reported in tumour tissues. Remarkably, our data showed that the cell lines analyzed here resemble most of the important genomic alterations previously described in primary HNSCC. It also revealed the presence of several regions with high level focal amplifications (11q21-22.2, 18p11.31-p11.21, 19p13.2-p13.13, and 21q11) that have been previously identified in HNSCC [1,11].

Although rarely detected in solid tumors, high level amplification at 11q22-q23 has been described not only in HNSCC [12,13] but in many malignancies including glioblastomas, renal cell carcinomas, sarcomas, and cervical, lung and pancreatic cancers [14-19] thus suggesting that this region may harbor gene(s) that, when amplified, have an active role in tumorigenesis and/or cancer progression. *YAP* gene has been identified as a candidate target gene in 11q22 amplicon in several human cancers [20-22]. However, to date, no specific genes have been proposed as targets in HNSCC.

In the present report, we performed gene expression analysis of the amplified genes in each amplicon identified in HNSCC-derived cell lines what allowed the identification of 12 novel genes with potential implications in HNSCC biology. One of the most dramatically amplified and overexpressed gene identified here is *TRPC6*, a member of the transient receptor potential (TRPC) subfamily, located at 11q22.1. This novel genetic change was also identified in primary HNSCC-tumour samples. Remarkably, recent studies have revealed that TRPC6 has an essential role in glioma growth, invasion, and angiogenesis [23,24]. We show here that *TRPC6* overexpression confers enhanced invasive behavior to HNSCC cells. Therefore, *TRPC6* may have an essential role in the development of the aggressive phenotype of HNSCC and may be a promising therapeutic target in the treatment of HNSCC.

## Methods

### Cell lines

The five established human HNSCC cell lines used in this study were kindly provided by Dr. Grenman [25]. Cell lines were derived from primary tumors located at the oral cavity (SCC2 and SCC40 cell lines) and larynx (SCC29, SCC38 and SCC42B cell lines). Cells were grown in Dulbecco’s modified Eagle’s medium supplemented with 10% fetal bovine serum, 100 units/ml penicillin, 200 μg/ml streptomycin, 2 mM L-glutamine, 20 mM Hepes pH 7.3 and 100 μM non-essential aminoacids. All cells were maintained at 37°C in 5% CO_2_.

### Tissue samples

Surgical tissue specimens from 24 patients with HNSCC were obtained, following institutional review board guidelines, from the Hospital Universitario Central de Asturias and Hospital General Universitario de Valencia. All the procedures utilized in this study are in agreement with the 1975 Helsinki Declaration. Informed consent was obtained from each patient. All the patients included in our study underwent surgical resection of their tumor and bilateral neck dissection (functional or radical based on surgical findings). All of them had a single primary tumor; none had undergone treatment prior to surgery, and had microscopically clear surgical margins. A portion of the surgical tissue specimen was sharply excised, placed in sterile tubes, and stored at −80°C in RNAlater (Ambion) for DNA and RNA analysis. Clinically normal adjacent mucosa and normal mucosa from non-cancer patients were also collected. All patients were habitual tobacco and alcohol consumers.

### DNA and RNA isolation

Genomic DNA was isolated using the QIAmp DNA Mini kit (Qiagen, Inc., Chatsworth, CA) and subsequently treated with RNase A (1unit/mL) at 37°C for 5 minutes. Total RNA was isolated from HNSCC cell lines and tumour tissues with Nucleospin RNA II (Macherey-Nagel, Easton, PA) following the manufacturer’s instructions with the addition of an extra acid phenol/chloroform extraction followed by RNA precipitation.

### Array-CGH

Arrays-CGH were performed as described by van den Ijssel et al. [26]. Briefly, tumour cell lines and reference DNAs (pooled from 10 different donors) were differently labelled by random priming. Three hundred ng test and reference DNA were hybridized to an array containing approximately 30,000 DNA oligos spread across the whole genome printed on Codelink activated slides (Amersham Biosciences, Barcelona, Spain). This array contained 29,134 oligos covering 28,830 unique genes. Hybridization and washing took place for two nights in a specialized hybridization chamber (GeneTAC/HybArray12 hybstation; Genomic Solutions/Perkin Elmer). Images were acquired using a Microarray Scanner G2505B (Agilent Technologies). Analysis and data extraction were quantified by BlueFuse (BlueGnome, Cambridge, UK). Gains were defined as at least two neighbouring oligonucleotides with deviations of 0.2 or more from log2 ratio = 0.0. High-level amplification was considered when at least two neighbouring clones reached a log2 ratio of 1.0 or higher.

### qPCR

Real-time PCR was done in an ABI Prism 7500 Real Time PCR System (Applied Biosystems, Foster City, CA) using Power SYBR Green PCR Master mix (Applied Biosystems) and the thermocycler conditions recommended by the manufacturer. Primers, designed using the computer program Primer Express (Applied Biosystems), were as described in Table 1.

**Table 1:** Oligonucleotides used for real time PCR

**Gene**	**Oligonucleotides**
*JRKL*	Forward: 5^′^CGCGATAGTCAGGGAGCTGT 3^′^
	Reverse: 5^′^GGGTTGGCTGGCAAATAGAC 3^′^
*CNTN5*	Forward: 5^′^CACCCCATCTCGAATGATCC 3^′^
	Reverse: 5^′^GGTGCTGTCTTCGGAACTGC 3^′^
*AD031*	Forward: 5^′^TCTCCTGTTGATTCGCAGATGT 3^′^
	Reverse: 5^′^ TTGAGACCAGTTGATGAATACTCGA 3^′^
*PGR*	Forward: 5^′^AACTTCTTGATAACTTGCATGATCTTG 3^′^
	Reverse: 5^′^AGCAGTACAGATGAAGTTGTTTGACA 3^′^
*TRPC6*	Forward: 5^′^TTCTCATGGATGGAGATGCTCA 3^′^
	Reverse: 5^′^CCATATCATGCCTATTACCCAGGA3^′^
*YAP1*	Forward: 5^′^GACTTCCTGAACAGTGTGGATGAG 3^′^
	Reverse: 5^′^TGCTTTGGTTGATAGTATCACCTGTAT 3^′^
*BIRC3*	Forward: 5^′^CATCCGTCAAGTTCAAGCCA 3^′^
	Reverse: 5^′^GATAGCAGCTGTTCAAGTAGATGAGG 3^′^
*PORIMIN*	Forward: 5^′^TGCTTCATCAGTAACAATCACAACA 3^′^
	Reverse: 5^′^CCTTTCTTTGCTTCAGAATGCAT 3^′^
*MMP7*	Forward: 5^′^CCAGGATGATATTAAAGGCATTCA 3^′^
	Reverse: 5^′^TGAATTACTTCTCTTTCCATATAGTTTCTGA 3^′^
*MMP20*	Forward: 5^′^CTGCTCTTCAAGGACCGGATT 3^′^
	Reverse: 5^′^TGTCCGCAAGTGAACCTGC 3^′^
*MMP27*	Forward: 5^′^GCATTTGGTGCTGGAGGTTT 3^′^
	Reverse: 5^′^ACCCTTTGTCCATGGTTTGG 3^′^
*MMP8*	Forward: 5^′^AGTTGATGCAGTTTTCCAGCAA 3^′^
	Reverse: 5^′^GGTCCACTGAAGACATGGAAGAA 3^′^
*MMP10*	Forward: 5^′^TGCATCAGGCACCAATTTATTC 3^′^
	Reverse: 5^′^GAGTGGCCAAGTTCATGAGCA 3^′^
*MMP1*	Forward: 5^′^TGGACCAACAATTTCAGAGAGTACA 3^′^
	Reverse: 5^′^TTCATGAGCTGCAACACGATG 3^′^
*MMP3*	Forward: 5^′^TCTTTGTAGAGGACAAATACTGGAGATT 3^′^
	Reverse: 5^′^CCATGGAATTTCTCTTCTCATCAA 3^′^
*MMP12*	Forward: 5^′^CGATGAGGACGAATTCTGGAC 3^′^
	Reverse: 5^′^CAGTGAGGAACAAGTGGTGCC 3^′^
*MMP13*	Forward: 5^′^GCCATTACCAGTCTCCGAGG 3^′^
	Reverse: 5^′^GCAGGCGCCAGAAGAATCT 3^′^
*RNMT*	Forward: 5^′^GTTCCTGAATTCTTGGTCTATTTTCC 3^′^
	Reverse: 5^′^CTTCTTTGCCATTTCATTTAGCAAT 3^′^
*MC5R*	Forward: 5^′^TTGGATCTCAACCTGAATGCC 3^′^
	Reverse: 5^′^TTGACATTGGGTCCTGAAAGG 3^′^
*MC2R*	Forward: 5^′^CCTTCTCATTCATTTTGCCCA 3^′^
	Reverse: 5^′^TCCCAATCACCTTCAGCTCG 3^′^
*ZNF443*	Forward: 5^′^GAACCTGGATTGTGTAGTAATGAAATG 3^′^
	Reverse: 5^′^TGATCTTCAATGTTCTGGTCTTTCC 3^′^
*MAN2B1*	Forward: 5^′^GCTCAAAACCGTGGACCAGT 3^′^
	Reverse: 5^′^GGCGTGCTGGATGTCATTCT 3^′^
*JUNB*	Forward: 5^′^AAACTCCTGAAACCGAGCCTG 3^′^
	Reverse: 5^′^CGCTTTGAGACTCCGGTAGG 3^′^
*STCH*	Forward: 5^′^AACCCGAGCAATGTCTGGAA 3^′^
	Reverse: 5^′^TGATTGAAGTCCTGTCCTCCAA 3^′^
*NRIP1*	Forward: 5^′^GGGATCAGGTACTGCCGTTG 3^′^
	Reverse: 5^′^TCCTCTTCATTATGCCCAGCA 3^′^
*CYPA*	Forward: 5^′^CATCTGCACTGCCAGACTGA 3^′^
	Reverse: 5^′^TTGCCAAACACCACATGCTT 3^′^

To perform mRNA quantifications, first-strand cDNA was synthesized from 2 μg of total RNA using the Superscript first-strand synthesis system for reverse transcriptase (Invitrogen, Carlsbad, CA) with random primers and oligodT according to the manufacturer’s directions. Cyclophilin was used to normalize for RNA input amounts and to perform relative quantification. To perform genomic DNA amplification, tyrosine hydroxylase gene was used to normalize for DNA input amounts and to perform relative quantification. Melting curve analysis showed a single sharp peak with the expected *T*_m_ for all samples and genes tested. Relative quantities were obtained using the 2^–ΔΔCt^ method [27].

### Western blot

Protein extracts were obtained from SCC42B cells at 70% to 80% confluence by scraping on ice in lysis buffer containing 50 mmol/l HEPES (pH 7.9), 250 mmol/l NaCl, 5 mmol/l EDTA, 0.2% NP40, 10% glycerol, and protease inhibitors (0.5 mmol/l phenylmethylsulfonyl fluoride, 1 μg/ml aprotinin, 10 μg/ml leupeptin and 1 mmol/l Na_3_VO_4_). Equal amounts of proteins were fractionated on SDS-PAGE and transferred to PVDF membranes. Membranes were probed with anti-TRPC6 antibody (Abcam) or anti-β-actin (Sigma-Aldrich) at 1:100 and 1:5000 dilutions, respectively. Bound antibodies were detected using Enhanced Chemiluminescence Reagent (Amersham Pharmacia Biotech) according to the protocol of the manufacturer.

### siRNA treatment

siRNA duplex oligonucleotides (ON-TARGETplus SMARTpool Human TRPC6) were purchased from Dharmacon Research (Lafayette, CO). siCONTROL Non-targeting pool (Dharmacon) were used as control siRNA. SCC42B cells were transfected with 35 pmol/ml siRNAs using Lipofectamine 2000. *TRPC6* mRNA analyses revealed a substantial inhibition (more than 60–70%) of *TRPC6* expression 48–72 hours after transfection. The transfected cells were used for subsequent experiments within that interval of time.

### Wound healing assay

Cells were grown to confluence in 35-mm tissue culture dishes. Cell monolayers were wounded using a micropipette tip, and floating cells were removed by extensive washing with DMEM. Photographs of the wounded area were taken immediately after making the scratch (0 h time point) and after 8 h using a Leica DMIL microscope to measure the migration rate of cells into the wounded area. At least 15 different fields were randomly chosen across the wound length. For the analysis of the differential cell migration capacity of SCC38, SCC40, and SCC42B cells, the rate of front migration of cell monolayers was analyzed in an AxioObserver.Z1 microscope (Zeiss), equipped with an incubation module, by taking pictures at 0 h and 8 h using an EC Plan-Neofluor 10x/0.30 Ph1 objective.

### Matrigel invasion assays

In vitro invasion assays were performed by using a 24-well invasion chamber coated with Matrigel (Becton Dickinson). Cells were trypsinized, washed with PBS, suspended in DMEM containing 5% BSA, and plated in the invasion chamber (3 x 10^4^ cells per well). The lower chambers were filled with DMEM containing 5% BSA with 10% FBS. After 24 h, the cells remaining in the upper chamber were removed by scraping, whereas the cells that invaded through Matrigel were fixed and stained by using 0.5% Crystal Violet in methanol. All invading cells were counted by microscopic visualization. All analyses were performed in triplicate.

### MTS-based cell proliferation assay

MTS assays were performed using CellTiter 96 Cell Non-Radioactive Proliferation Assay following the protocol recommended by the manufacturer (Promega, Madison, WI). Briefly, 1000 cells were seeded in each well of 96-well plates, and allowed to growth for 48, 72 or 96 hours. MTS assay was performed at each time point.

## Results and discussion

### Array CGH analysis of HNSCC-derived cell lines

**Figure 1:**
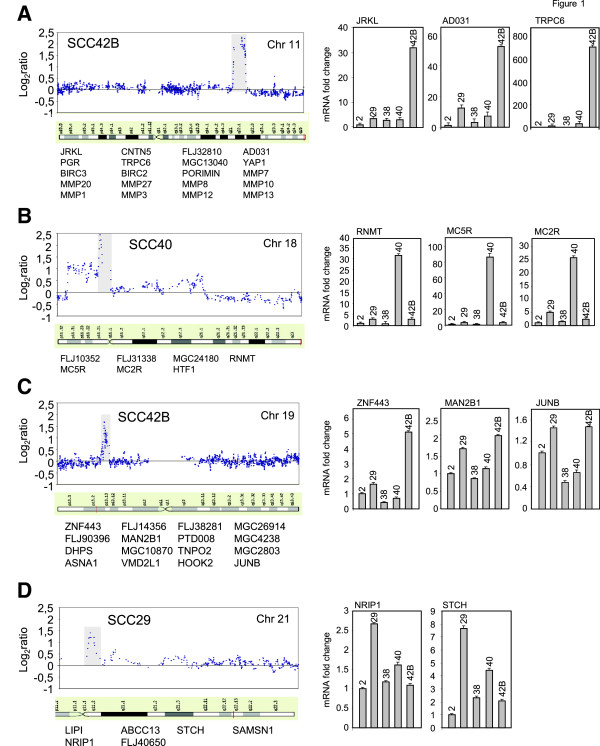
**Genome-wide copy number plots of gene amplifications and relative mRNA expression data in HNSCC-derived cell lines.** Left panels show the profiles as normalized log2 signal intensity ratios of each spot on the array to the genomic position at chromosome 11 (**A**), chromosome 18 (**B**), chromosome 19 (**C**) from p-to t-telomere, and chromosome 21 (**D**) from chromosomal band 11p11.2 to t-telomere. Right panels show the relative mRNA levels of the indicated genes in the HNSCC-derived cell lines. Total RNA was extracted from HNSCC-derived cell lines grown to 80–90% confluence. mRNA levels were analyzed by RT-qPCR.

Array CGH was used to characterize genome-wide DNA copy number alterations in five HNSCC-derived cell lines. Visual inspection of the array CGH profiles revealed the presence of an overall pattern that is broadly consistent with the literature in HNSCC (a summary of the chromosomal aberrations is shown in Table 2). Some degree of gain and/or loss was detected in every cell line. The data predicted frequent copy number gains (present in three or more cell lines) for specific segments in 3q, 5p, 7p, 8q, 9q, 11q, 14q, 18p, and 20q; and losses for 3p, 9p, 11q, and 18q. These copy number alterations, revealed through CGH-array, had been previously detected with conventional metaphase CGH analysis in HNSCC primary samples [1,28]. High-level amplifications were detected at four chromosomal sites including 11q21-q22.2, 18p11.31-p11.21, 19p13.2-p13.13, and 21q11 (see Figure 1). Gains encompassing these genomic regions have been described in previous reports [11,12,29,30]. In addition to known regions, our CGH-array analysis disclosed alterations that had never been reported using conventional techniques, such as small gains in 4p12, 13q12, 21q21, and losses in 22q13 (Table 3).

**Table 2:** Most frequently reported chromosomal gains and losses present in HNSCC-derived cell lines

**Chro**	**Region**	**Size (Mb)**	**Frequency**	**Known proto-oncogenes**	**Cell line with minimal region of change**
*Chromosomal gains*					
1p	1p32.1-p21.1	47,25	2/5	-	SCC40
3q	3q13.2-qter	84,9	4/5	BCL6, EIF4A2, EVI1, GMPS, LPP, MDS1, MLF1, PI3K3CA, RPN1, TFRC, ZNF9	SCC2
5p	5pter-p12	45	3/5	LIFR	SCC38
6q	6q16.3-q23.3	38,82	1/5	FOXOA3, GOPC, ROS1, STL	SCC40
7p	7pter-p14.3	32	3/5	ETV1, HOXA9, HOXA11, HOXA13, HNRPA2B1, JAZF1, PMS2	SCC29
7q	7q21.13-q31.1	22,87	1/5	AKAP9, CDK6	SCC2
8q	8q21.1-q24.22	64,6	5/5	COX6C, EXT1, MYC, NBS1	SCC29
9p	9p21.2-p13.2	12,28	2/5	PAX5, FANCG	SCC29
9q	9q21.33-q34.11	43	4/5	FANCC, NR4A3, OMD, PTCH1, SYK, TAL2, XPA	SCC29
11q	11q12.2-q12.3	1,9	4/5	-	SCC42B
	11q13.2-q22.2	33,1	4/5	PRAD1, NUMA1, PICAM, MAML2, BIRC3	SCC40
	11q23.3	0,20	3/5		SCC40
				DDX6	
14q	14q23.1-q24.2	13,23	1/5	GPHN, RAD51L1	SCC2
	14q31.1	0,72	3/5	TSHR	SCC2
18p	18p11.31-p11.21	13.37	4/5	-	SCC40
19p	19p13.2-p13.13	1,64	1/5	LYL1	SCC42B
20q	20q11.21-q11.23	4,86	3/5	-	SCC42B
*Chromosomal losses*					
1p	1p13.2-p12	7,35	2/5	NRAS, TRIM33	SCC29, SCC40
3p	3p23-p22.3	2,5	4/5	MLH1	SCC2
5q	5q11.1-q12.3	13,11	2/5	-	SCC29, 38
8p	8pter-q11.21	47,1	2/5	PCM1, FGFR1, WRN, WHSC1L1	SCC40
9p	9p21.3	2,61	3/3	CDKN2A, CDKN2B, MLLT3	SCC29
10p	10pter-p11.21	37,35	2/5	COPEB, MLLT10, SH3BP1	SCC2, SCC40
11q	11q22.3-qter	15,29	3/5	ATM, CBL, DDX10, PAFAH1B2, POU2AF1, SDHD, ZNF145, FLI1, PRO1073	SCC42B
18q	18q21.1-qter	29,97	3/5	BCL2, FVT1, SMAD4, MALT1	SCC40
22q	22q11.21	1,03	2/5	BCR, CLTCL1, PNUTL1, SMARCB1	SCC29
	22q12.1-q12.2	1,96	2/5		SCC40

**Table 3:** Non previously identified altered chromosomal regions

**Chro**	**Alteration**	**Region**	**Size (Mb)**	**Frequency**	**Known proto-oncogenes**	**Cell line with minimal region of change**
4	gain	4p12	7,08	1/5	TEC	SCC29
13	gain	13q12.12-q12.3	5,49	2/5	CDX2, FLT3	SCC29, SCC40
21	amplification	21q11	1,20	1/5	-	SCC29
	gain	21q21.1	1,36	5/5	-	SCC29, SCC40
	gain	21q21.3	4,59	2/5	-	SCC29, SCC38
22	loss	22q13.2	0,62	5/5	-	SCC2, SCC29

In general, the array CGH data showed that the recurrent genome aberrations described in primary HNSCC tissues are well preserved in the cell lines analyzed here. It also indicates that these cell lines have not accumulated substantial novel recurrent aberrations during extended culture. These data, together with our previous molecular and functional studies [31,32], suggest that analysis of genomic aberrations in the HNSCC-derived cell lines used here might be a useful approach to identify tumor-associated chromosomal regions substantial for the tumorigenesis and progression of HNSCC.

### Impact of focal high-level amplifications on gene expression

To gain some insights into the role of genomic aberrations in HNSCC pathophysiology, we focused in focal amplification events for which it may be easier to pinpoint target genes involved in the pathogenesis of HNSCC.

The present analysis allowed narrowing down and delineating the boundaries of high-level amplification events. Boundaries from the p-telomere span from 95 to 102 Mb (11q21-q22.2), 3,44 to 16,81 Mb (18p11.31-p11.21), 11 to13 Mb (19p13.2-p13.13), and 14,1 to 15,3 Mb (21q11). These are relatively small genomic segments containing 20 or fewer genes (listed in Figure 1) suggesting that any of them may be the target(s) of the amplification. These amplicons do not contain well-established oncogenes in HNSCC. To identify putative driver genes in these genomic regions, we compared the expression levels of candidate genes mapping in the amplicons with their DNA copy number status. Figure 1 illustrates genome-wide copy number plots of the gene amplifications and the gene expression data.

Interestingly, a high degree of correlation between DNA and mRNA levels was found for most of the genes selected at 11q, 18p, 19p, and 21q amplicons. This is in agreement with previous studies showing that amplification has a strong impact on transcription levels [33-35]. Expression of *RNMT*, *MC5R*, and *MC2R* genes at 18p11.31-p11.21 amplicon was significantly up-regulated in SCC40 cells that had shown high-level amplification at that locus, compared with cell lines without gene amplification (p < 0,0001) (Figure 1B). Similarly, the expression levels of the *STCH* and *NRIP1* genes at 21q11 were significantly higher in SCC29 cells, which harbored amplification at that locus, than in the other cell lines without gene alteration (p < 0,01) (Figure 1D). Amplification of the *ZNF443*, and *MAN2B1* genes at 19p13.2-p13.13, detected in SCC42B cells, also correlated with higher expression at the mRNA levels as compared with the other cell lines (p < 0,05) (Figure 1C). However, quantification of the mRNA levels of the *JUNB* proto-oncogene (19p13.2-p13.13) revealed that SCC42B cells had similar levels of expression than SCC29 cells, which did not show amplification of the 19p13.2-p13.3 locus. These data indicate that *ZNF443* and/or *MAN2B1* genes, but not *JUNB*, might be candidates of the selection pressure for structural amplification of the 19p13.2-p13.3 region, at least in SCC42B cells. In general, any of the amplified and over-expressed genes identified here (*RNMT, MC5R, MC2R, ZNF443, MAN2B1, NRIP1,* and *STCH*) might be up-regulated in a DNA copy number-dependent manner and could possibly contribute to HNSCC pathogenesis. To our knowledge, no previous evidence is available on the association of these genes in HNSCC biology. Of all the genes analyzed here, only *JUNB* has been previously found up-regulated at the mRNA and protein level in HNSCC tumour tissues [36-39]. Our data suggest that its over-expression is caused by mechanisms other than gene amplification. Nevertheless, further studies are required to demonstrate unequivocally whether an association exists between the genetic and expression data in tumour tissue samples.

With regard to the 11q21-q22.2 amplicon, recent studies reported high copy number amplification at this locus in HNSCC [12,13,30]. This region contains 18 known genes harbouring two gene clusters, one with nine matrix metalloproteinase (*MMP*) genes, and other with two baculoviral IAP repeat-containing protein (*BIRC*) genes. Expression analysis of *BIRC* and *MMP* genes in the HNSCC-derived cell lines showed no correlation between their mRNA levels and DNA copy number status. In contrast, expression of *JRKL*, *AD031*, *TRPC6*, (Figure 1A), *YAP1* and *PORIMIN* (data not shown) genes were significantly up-regulated in SCC42B cells that had shown high-level amplification at that locus, compared with cell lines without gene amplification (p < 0,01). Specifically, mRNA levels of *JRKL*, *AD031*, *TRPC6*, *YAP1*, and *PORIMIN* were, respectively, 30, 50, 600, 10, and 8-fold higher in SCC42B cells than in the other cell lines. mRNA expression of other candidate genes at 11q21-q22.2 amplicon (*CNTN5, PGR*, and *MMP27*) was not detected in any of the cell lines. These data exclude *CNTN5, PGR*, *MMP* and *BIRC* genes and point to any of the 5 amplified and over-expressed genes as critical gene-amplification “driver/s”. Of them, only *TRPC6* and *YAP1* genes have been previously found deregulated in several types of cancer. Amplification and mRNA up-regulation of *YAP1* has been previously described in several cancers including HNSCC of the oral cavity [20,30,40], sarcomas, meduloblatomas, and mesotheliomas [20,21,41,42]. In addition, recent studies showed that over-expression of *YAP1* induces phenotypic alterations that are commonly associated with potent transforming oncogenes [40,42-44]. *TRPC6* is a member of the TRP family of Ca^2+^- and Na^+^-permeable channels shown to be up-regulated in glioblastomas and breast, prostate, gastric, and oesophageal cancer cells [23,45-48]. Our data revealed that this was the most dramatically up-regulated gene in SCC42B cells. However, to the best of our knowledge, up-regulation of *TRPC6* has not been previously identified in HNSCC.

### *TRPC6* gene is amplified and over-expressed in HNSCC-tissue specimens

*TRPC6* DNA and mRNA levels were analyzed in a panel of 24 primary tumors (Table 4). Eight out of 24 tumor samples displayed increased gene copy number as compared with a pool of DNA samples obtained from normal mucosa of five healthy individuals. Analysis of *TRPC6* mRNA levels revealed that it was absent in normal mucosa from non-cancer patients. Similarly, it was either absent or barely detectable in all clinically normal mucosa adjacent to tumors, and in 11/24 tumor samples. In contrast, 13 tumor tissues displayed *TRPC6* mRNA levels that were 1.7- to 19-fold above the highest level found in normal mucosa. All but one tumor showing increased *TRPC6* gene dosage also harbored *TRPC6* mRNA over-expression. These data suggest that *TRPC6* amplification may be responsible for *TRPC6* over-expression and is a candidate driver gene in 11q21-q22.2 amplicon that may play a role in HNSCC pathophysiology.

**Table 4:** **Relative *TRPC6 *DNA and mRNA levels in HNSCC primary tumors**

**Tumor sample**	Genomic *TRPC6 *DNA levels^*^	*TRPC6 *mRNA Levels^*^
7 T	**5.70**	**1.7**
8 T	0.67	1.2
11 T	**2.01**	**2,85**
12 T	1.60	1.38
13 T	**3.90**	**1.9**
14 T	0.60	0.56
17 T	**2.00**	1.01
21 T	**3.00**	**5.77**
23 T	1.27	**7.5**
25 T	1.27	1.05
26 T	1.75	**5.91**
27 T	1.55	**1.84**
32 T	**2.60**	**1.7**
33 T	1.40	**9.53**
95 T	0.37	1.46
110 T	1.60	1.38
112 T	**2.90**	**4.14**
124 T	0.80	**2.87**
127 T	0.86	**1.76**
141 T	**3.59**	**19.02**
143 T	0.60	1.03
147 T	0.60	0.16
154 T	1.40	0.41
155 T	0.61	0.16

^*^Values showing gene gain (#2) and increased mRNA levels (#1.7) are indicated in bold.

### Inhibition of *TRPC6* expression does not induce changes in SCC42B cell proliferation

**Figure 2:**
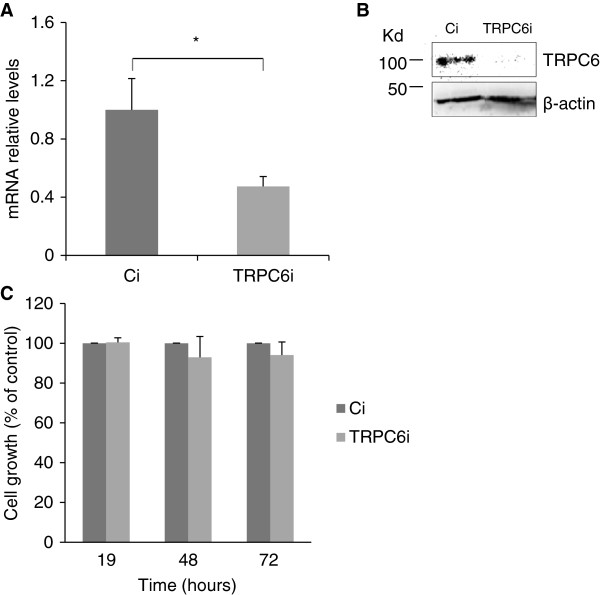
**TRPC6 inhibition does not affect cell proliferation in SCC42B cells.** SCC42B cells were transfected with control (Ci) or TRPC6-siRNA (TRPC6i) 48 hours before MTS assay. (**A** and **B**) Reduction of TRPC6 mRNA (**A**) and protein (**B**) levels by siRNA treatment. Transcripts were quantified using RT–qPCR. The mean of relative expression to cyclophilin A housekeeping gene of at least three independent experiments is shown. (**C**) Cell growth was determined using a colorimetric MTS assay. Columns, mean cell growth relative to control of three independent experiments. * p < 0.05 paired Student’s *t* test.

Previous studies have shown that inhibition of *TRPC6* expression results in decreased cell proliferation in cancer cells [23,24,47,49,50]. To investigate the possible role of *TRPC6* on cell proliferation of HNSCC cells, MTS assays and cell counting were performed in SCC42B cells expressing siRNA against *TRPC6*, and in their corresponding control cells. As shown in Figure 2, inhibition of *TRPC6* expression did not affect significantly the cell growth rates. Accordingly, the number of cells in each phase of the cell cycle was similar in SCC42B cells transfected with *TRPC6* siRNA versus control siRNA (data not shown). We did not find association between the proliferation rate of SCC cells and the presence of *TRPC6* gene amplification and over-expression. SCC42B cells carrying 11q21-q22.2 amplification proliferate more rapidly than SCC29 and SCC40 cells, but they growth at similar rates than SCC38 and SCC2 cells (data not shown). These data show that, in the tumour background examined here, *TRPC6* is not important for cell proliferation.

### Inhibition of *TRPC6* expression impairs cell migration and invasion

**Figure 3:**
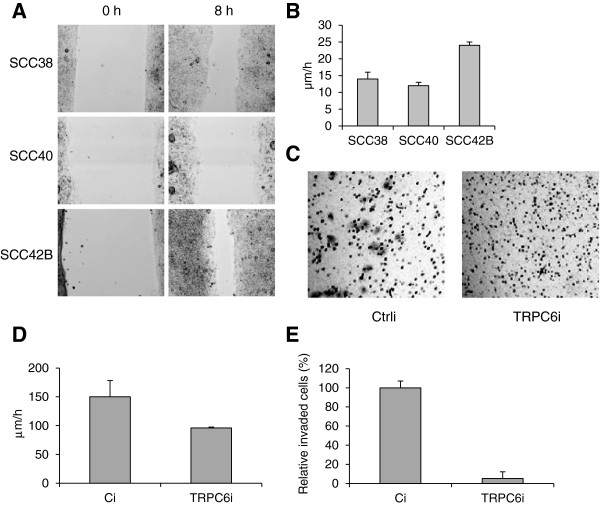
**Inhibition of TRPC6 gene expression decreases cellular migration and invasion.** (**A** and **B**) Wound healing assays were performed in SCC38, SCC40 and SCC42B cells. The rate of front migration of cell monolayers was analyzed by time-lapse video microscopy. At least 15 different fields were randomly chosen across the wound length. Values are mean of average ± s.d. from three independent experiments. (**C** and **E**) SCC42B cells treated with control (Ci) or TRPC6 siRNA (TRPC6i) were seeded in serum-free media in the upper chamber of Matrigel transwells. The lower chamber was loaded with regular media supplemented with 10% fetal bovine serum and 5% BSA. After 24 h at 37°C in 5% CO_2_, the top filter was scraped, and invading cells were fixed and stained. (**C**) Representative images captured with a 10 objective 24 h after seeding. (**E**) All invading cells were counted under x10 magnification. Values are mean of average ± s.d. from three independent experiments done in triplicate. (**D**) Inhibition of TRPC6 expression in SCC42B cells attenuates cell migration. Wound healing assays were performed in cells treated with TRPC6- (TRPC6i) or control-siRNA (Ci). Values are mean of average ± s.d. from three independent experiments.

In addition to cell proliferation, Ca^2+^ signaling is known to be involved in cell locomotion. It was therefore tempting to speculate that SCC42B cells have a high migratory capacity. Comparison of the cell migration behavior of SCC38, SCC40 and SCC42B cells revealed that the migratory potential of SCC42B cells, which express high levels of *TRPC6* and harbor 11q21-q22.2 amplification, was significantly higher than that of SCC38 and SCC40 cells, containing lower levels of *TRPC6* mRNA and genomic DNA (Figure 3A and B). This different phenotype may be the result of different levels of TRPC6 gene expression or, alternatively, could be caused by other gene(s)/protein(s) structural or functional alterations in the cell lines explored here. We therefore sought to determine whether inhibition of *TRPC6* expression by siRNAs affects cell migration in SCC42B cells. As shown in Figure 3D, knock down of *TRPC6* expression by siRNA resulted in a 36% decrease in cell migration as compared with cells transfected with nonspecific siRNAs. SCC42B cells were also analyzed for their invasive potential through a B1-mm Matrigel barrier compared with cells transfected with TRPC6 siRNA. The data revealed that invasion was dramatically inhibited with TRPC6 siRNA expression showing a ~90% decrease in invasiveness (Figure 3C and E).

Plasma membrane ion channels contribute to virtually all basic cellular processes and are also involved in the malignant phenotype of cancer cells by modulating different hallmarks of cancer such as proliferation, cellular locomotion, and tissue invasion. Specifically, the morphological and adherence changes of metastatic cells involve Ca^2+^ signaling supported by enhanced Ca^2+^ influx. Recently, TRPC6 has emerged as an important player in the control of the aggressive phenotype of glioblastoma cells [23]. Our analysis of the functional significance of TRPC6 overexpression in HNSCC showed that TRPC6 also modulates cell invasion in HNSCC cells. This finding is of interest as it provides the opportunity to therapeutically target TRPC6 to interfere with Ca^2+^-dependent signaling involved in cell invasion.

## Conclusions

In the present study, we report that *TRPC6* (11q22) is overexpressed in HNSCC, and provide new evidence that increase in gene dosage is a novel mechanism to activate *TRPC6* expression in cancer. Increased *TRPC6* mRNA and gene dosage was detected in both, cell lines and tumor tissues, revealing that this molecular alteration can be pathologically relevant in HNSCC. In addition, siRNA-induced knockdown of *TRPC6* expression in HNSCC-derived cells dramatically inhibited HNSCC-cell invasion. Therefore, *TRPC6* is likely to be a target for amplification that confers enhanced invasive behavior to HNSCC cells and, therefore, may be a promising therapeutic target in the treatment of HNSCC. These data provide the foundation for further functional validation of this putative candidate gene in tumor tissues to determine whether it is crucial for tumor development or progression.

## Competing interests

The authors declare that they have no competing interests.

## Authors’ contributions

SBQ and AM carried out the functional assays and the molecular genetic studies. PS and IZ carried out the gene expression studies. ISS and ND participated in the invasion assays. CS, EJ and RS participated in the acquisition of the data and performed the statistical analysis. MDC conceived of the study, participated in its design and coordination, and drafted the manuscript. All authors read and approved the final manuscript.

## Pre-publication history

The pre-publication history for this paper can be accessed here:

http://www.biomedcentral.com/1471-2407/13/116/prepub
